# The immediate effect of acupuncture on brain function in patients with chronic itch: a study protocol for an fNIRS investigation

**DOI:** 10.3389/fmed.2025.1726261

**Published:** 2025-12-31

**Authors:** Chang’en Peng, Xinglin Liu, Huijing Li, Haiyan Qin, Dongling Zhong, Xiaobo Liu, Yuting Dong, Rongjiang Jin, Xianjun Xiao, Juan Li

**Affiliations:** 1 Chengdu Pidu District Hospital of Traditional Chinese Medicine, Chengdu, China; 2 School of Management, Chengdu University of Traditional Chinese Medicine, Chengdu, Sichuan, China; 3 College of Clinical Medicine, Chengdu University of Traditional Chinese Medicine, Chengdu, Sichuan, China; 4 The First Affiliated Hospital of Shenzhen University, Shenzhen, Guangdong, China; 5 School of Health Preservation and Rehabilitation, Chengdu University of Traditional Chinese Medicine, Chengdu, Sichuan, China; 6 Affiliated Sichuan Provincial Rehabilitation Hospital of Chengdu University of Traditional Chinese Medicine, Chengdu, Sichuan, China

**Keywords:** acupuncture, chronic itch, near-infrared spectroscopy, study protocol, randomized controlled trial

## Abstract

**Background:**

Chronic itch significantly affects patients’ quality of life. Acupuncture shows therapeutic potential for chronic itch, while its neural mechanisms remain unclear. This study aims to investigate the immediate anti-itch effects of acupuncture in patients with chronic itch and explores the underlying neural mechanisms through functional near-infrared spectroscopy (fNIRS) monitoring.

**Methods:**

This study is a randomized controlled design. A total of 40 participants with chronic itch will be randomly assigned to the verum acupuncture group or the sham acupuncture group. The fNIRS will be used to monitor hemodynamic activity and functional connectivity of the prefrontal cortex and motor areas. The data will be continuously acquired across three consecutive 5-min epochs: (1) pre-stimulation baseline (5 min before needle insertion), (2) intra-stimulation phase (during 5-min needle retention), and (3) post-stimulation observation (5 min immediately after needle withdrawal). The primary outcome is the change in itch numeric rating scale score, secondary outcomes include the degree of itch relief, immediate comfort, intensity of itch, deqi intensity scale and expectation of therapeutic effect. In addition, both regional brain activation and functional connectivity will be analyzed.

**Discussion:**

This study plans to observe the immediate anti-itch effects of acupuncture for patients with chronic itch and reveal associated brain functional changes using fNIRS. The findings will provide a neurobiological basis for the anti-pruritic mechanisms of acupuncture.

**Clinical trial registration:**

http://itmctr.ccebtcm.org.cn/, identifier ITMCTR2025001506.

## Introduction

Itch, an unpleasant sensory experience, fundamentally arises from the activation and central processing of specific neural pathways in the brain (1, 2). Chronic itch is defined as itch that persists for more than 6 weeks (3). The 2023 Global Burden of Disease Database indicates that approximately 10.84 million individuals worldwide are affected by pruritus-related diseases (4). A cohort study by Matterne et al. demonstrated that the prevalence of chronic itch can be as high as 25% (5). Chronic itch commonly causes emotional distress, sleep disturbances, and difficulties in performing daily activities, thereby substantially compromising patients’ mental and social well-being as well as their overall quality of life (6–8). The pathogenesis of chronic itch involves dysregulated signaling across multiple systems, ranging from the skin barrier and immune cells to peripheral nerve fibers, spinal cord, and brain regions (9). Currently, no medications are Food and Drug Administration approved specifically for the treatment of chronic itch (10). The conventional management of chronic itch in clinical practice relies on symptom-targeted agents such as antihistamines, corticosteroids, and immunosuppressants (11). Nevertheless, these approaches are associated with notable limitations, including suboptimal efficacy and significant adverse effects from prolonged administration (11), Thus driving the need for novel therapeutic strategies.

Acupuncture has been widely applied in the management of itch (12–15). Meta-analysis of Wei et al. has shown that acupuncture resulted in a greater reduction in itch scores in comparison to loratadine or sham acupuncture controls (16). Du et al. reported that acupuncture combined with bloodletting therapy was significantly more effective than oral loratadine in reducing itch (17). In a meta-analysis, Lu et al. found that itch scores in the acupuncture plus traditional Chinese medicine group were significantly lower than those in the conventional western medicine group (18). Similarly, the research conducted by Shi et al. indicated that acupuncture therapy was significantly more effective than oral cetirizine hydrochloride in alleviating patients’ itching symptoms (13). Our previous studies also demonstrated that acupuncture significantly alleviated itch in patients with chronic spontaneous urticaria (19, 20). Although the sustained therapeutic effects of acupuncture have been documented, a critical knowledge gap remains: the immediate central effects of acupuncture during needling and their underlying cortical mechanisms in chronic itch patients are still poorly understood.

Chronic itch drives central sensitization, a state of neuronal hyperexcitability in the spinal cord and brain, leading to a heightened and exaggerated response to itch stimuli (21). Conversely, brain activity also influences chronic itch. In a mouse model of atopic dermatitis, Zhanmu et al. (22) established a role for the medial prefrontal cortex (mPFC) in itch processing, showing that kainic acid-induced lesions of this region significantly attenuated scratching behavior.

Neuroimaging modalities have been widely used to elucidate central mechanisms underlying itch, providing critical insights into cortical and subcortical processing of pruriceptive signals (23, 24). A functional magnetic resonance imaging (fMRI) study found that the functional connectivity of the right orbitofrontal cortex (OFC), medial prefrontal cortex, and premotor cortex in patients with chronic spontaneous urticaria was more deactivated than in healthy individuals (25). To investigate the mechanisms underlying itch sensation, Li et al. used near-infrared spectroscopy to monitor the dynamic changes of cortical oxygenated hemoglobin (HbO) and deoxygenated hemoglobin (HbR) concentrations in healthy subjects during histamine-induced itch, and the results demonstrated that multiple channels corresponding to Brodmann areas in the frontal cortex were activated (26).

Functional neuroimaging has greatly advanced our knowledge of itch-related brain activity, yet most studies rely on fMRI, which is costly, less tolerant to movement, and unsuitable for real-time monitoring during acupuncture. In contrast, functional near-infrared spectroscopy (fNIRS) provides a non-invasive, motion-tolerant, and temporally sensitive method for monitoring dynamic cortical hemodynamic changes during treatment (27, 28). This technique offers real-time monitoring, high time resolution and simple operation (29), and demonstrates robustness against motion interference and the ability to capture reliable data even during spontaneous itch (30).

Therefore, based on prior evidence (22, 25), we hypothesize that acupuncture’s immediate antipruritic effect correlates with modulation of cortical regions involved in itch processing, particularly the mPFC, OFC, and premotor cortex, which can be captured in real-time by fNIRS. The term “immediate effect” refers to the rapid, short-term modulation of symptoms observed shortly after a single acupuncture intervention. Thus, this study aims to characterize acupuncture’s immediate effect and corresponding cortical response using fNIRS technology.

## Methods

### The study design

This prospective randomized controlled trial will be conducted at the Pidu District Hospital of Traditional Chinese Medicine in Chengdu. Participants will be recruited through posters and the outpatient department of dermatology from August 1 to December 31, 2025. This trial has been approved by the Ethics Review Committee of Chengdu, Pidu District Traditional Chinese Medicine Hospital (K-2025-047), and registered on the http://itmctr.ccebtcm.org.cn/ (ITMCTR2025001506).

### Study population

#### Eligible criteria

Inclusion criteria are as followed: (1) Participants should have a clear dermatological diagnosis, such as chronic urticaria (31), atopic dermatitis (32), psoriasis (33), or neurodermatitis (34). (2) Right-handed participants, aged 18 to 60, with a minimum of a primary school education, with no restriction on gender. (3) Participants with a duration of itch ≥ 2 months and an average Numerical Rating Scale (NRS) score ≥ 4 over the past week, daily occurrence of itch, and relatively stable itch intensity over the preceding 2 weeks.

Exclusion criteria are listed below: (1) Participants with severe mental illness (e.g., schizophrenia, bipolar disorder) or cognitive impairment. (2) Participants who are administrated with any medication known to influence chronic itch symptoms in the 2 weeks prior to enrollment. (3) Participants with contraindications for acupuncture treatment (such as bleeding tendency or localized infection at acupoints, hemophilia). (4) Pregnant or lactating women; patients with skin infections or other acute dermatological conditions. (5) Participants with severe hepatic/renal dysfunction or cardiopulmonary diseases. (6) Participants with a history of alcohol abuse or substance use disorders. (7) Participants unable to cooperate with the fNIRS examination. (8) Participants who have received acupuncture treatment for itch within the past 6 months.

#### Sample size

Based on the observed change in itch NRS (numeric rating scale) scores from the pilot experiment (verum acupuncture group: 2.86 ± 1.07 VS sham acupuncture control group: 2.00 ± 0.58). With 80% statistical power, a 5% significance level, and accounting for a projected 20% dropout rate. Each group requires 20 people, and a total of 40 subjects need to be recruited. The calculation was performed using an *a priori* power analysis conducted with G*Power software (version 3.1), based on a t-test for the difference between two independent means (35).

#### Randomization and allocation concealment

**Figure 1:**
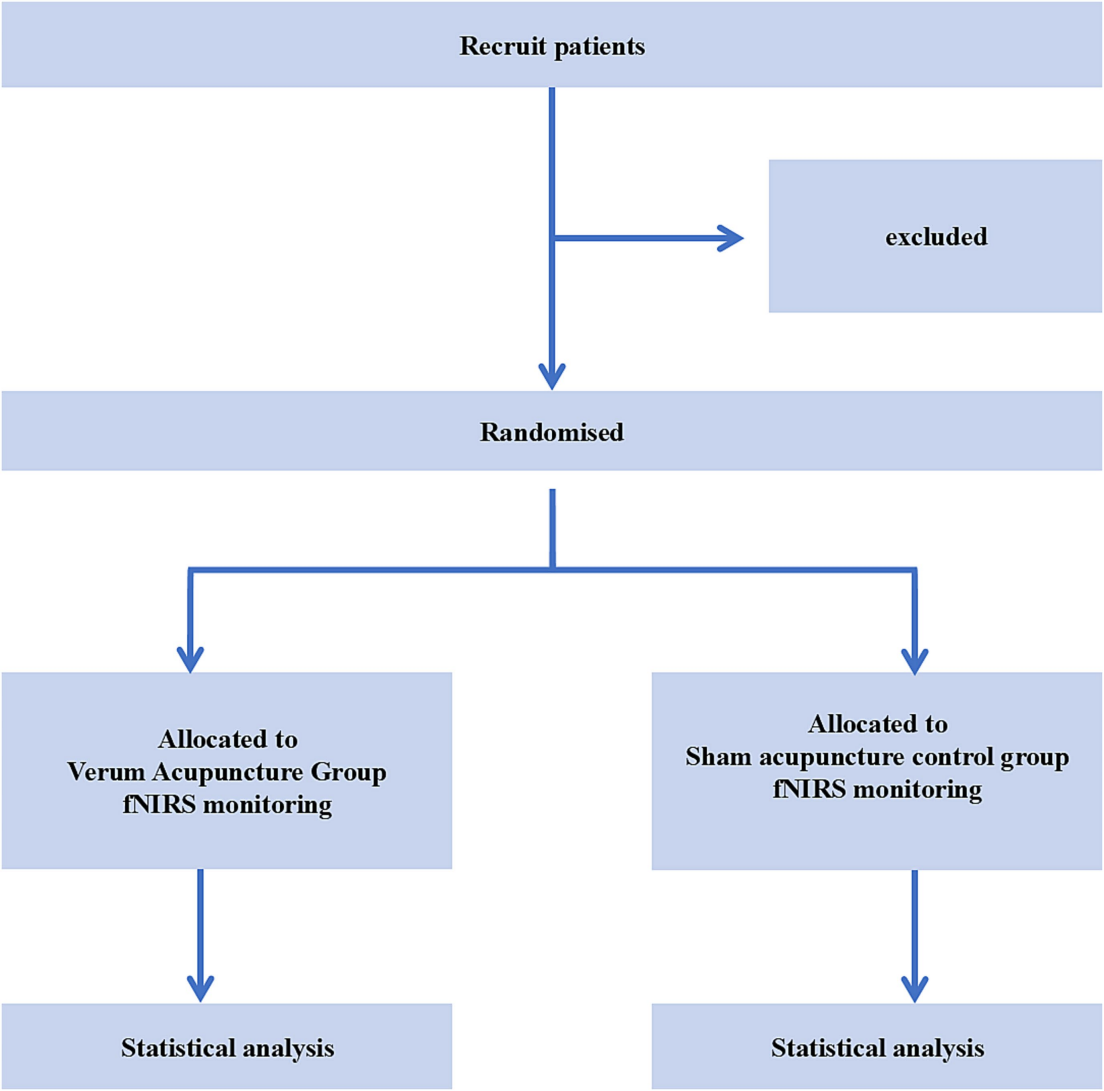
Flowchart of trial process.

The random numbers will be generated using SPSS 26.0 software. An independent research assistant not involved in recruitment or evaluation will place the random numbers sequentially into sealed, opaque envelopes. Each envelope is individually labeled with a unique consecutive serial number. Eligible patients will be randomly assigned to the verum acupuncture group and the sham acupuncture control group at a 1:1 ratio. The flowchart is shown in Figure 1.

#### Blinding

During the process of treatment, patients will wear opaque eye masks for all interventions to prevent visual identification of treatment type (verum acupuncture vs. sham acupuncture). Patients will complete a blinding assessment during the Post-Acupuncture Resting State phase. Acupuncturists will be unblinded to group assignment, and identical procedures will be maintained for both interventions. The evaluators will conduct a blind assessment to reduce expectation bias and observer bias in data collection. Statistical analyses will be conducted by a statistician blinded to group allocation.

### Intervention

#### Verum acupuncture group

**Figure 2:**
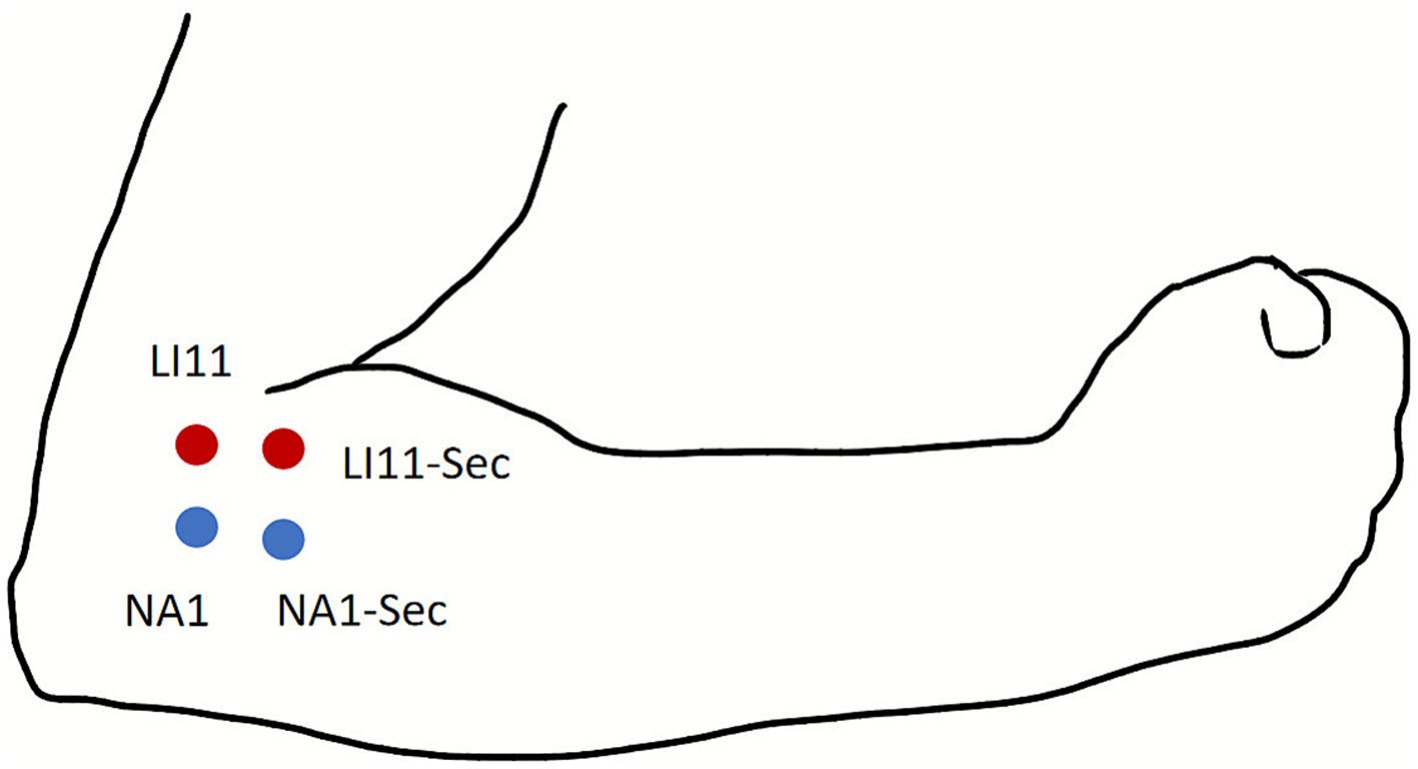
Locations of acupoints and non-acupoints. NA, non-acupoint.

Acupuncture will be performed at the Quchi acupoint (LI11) bilaterally using the following standardized protocol: Sterile, single-use acupuncture needles measuring 0.30 mm in diameter and 40 mm in length will be used. The needle will be inserted to a depth of 0.5 inches (approximately 1.25 cm). After achieving DeQi sensation with the primary needle, an auxiliary needle (0.18 mm in diameter and 13 mm in length) will be inserted adjacent to the primary needle to allow for the application of electrical stimulation. Electrical stimulation will be applied to both the primary needle and auxiliary needle for 5 min (20 Hz continuous wave, current intensity 1–5 mA). Acupoint locations are depicted in Figure 2.

#### Sham acupuncture group

The sham acupuncture procedure will be administered at non-acupoint location, specifically 1.0 cm inferior and lateral to LI11 bilaterally. Two blunt-tip needles (0.30 mm in diameter and 40 mm in length) will be applied: one at the designated non-acupoint (sham primary acupoint) and an auxiliary needle placed 0.5 cm adjacent to it (sham auxiliary point). All procedural protocols (needle retention time: 5 min, and electrode placement) will match the verum acupuncture group except for the use of a blunt-tip needle that does not penetrate the skin and the absence of electrical output. Acupoint locations are displayed in Figure 2.

### Outcomes

The assessment will be conducted before and after the treatment, and the assessment time points are presented in Table 1.

**Table 1:** Data collection schedule for clinical assessments.

Research phase	Pre-acupuncture resting state	Intervention period	Post-acupuncture resting state
Timepoint	5 min before needle insertion	During the needle retention period	5 min immediately after needle withdrawal
Medical history			
Signed informed consent	✓		
Inclusion/exclusion criteria	✓		
Demographic & baseline data			
Demographic questionnaire	✓		
Past medical history	✓		
Concomitant treatments	✓		
Psychometric scales			
Self-Rating Depression Scale	✓		
Pittsburgh Sleep Quality Index	✓		
Generalized Anxiety Disorder-7	✓		
Clinical assessments			
Degree of itch Relief		✓	
Itch intensity	✓	✓	✓
Immediate comfort level		✓	
Treatment expectancy	✓		
Treatment credibility scale		✓	
Deqi intensity scale		✓	
Trial evaluation			
Adverse events record			✓
Physician/Patient Global Assessment			✓
Blinding assessment			✓

#### Primary outcomes

The primary outcome was defined as the change of the itch NRS score, calculated by subtracting the post-intervention score from the baseline score. The intensity of itch will be evaluated using the NRS. The NRS is a patient-reported instrument in which subjects rate their subjective itch intensity on a numerical scale anchored at 0 (“no itch”) and 10 (“worst imaginable itch”). Measurements will be conducted at pre-acupuncture resting state, intervention period, and post-acupuncture resting state.

#### Secondary outcomes

##### Degree of itch relief

The degree of itch relief will be assessed immediately following each acupuncture treatment using a 5-point scale ranging from −2 to 2, where −2 indicates “significantly worsened,” −1 indicates “mildly worsened,” 0 indicates “no relief, 1 indicates “mildly relieved,” and 2 indicates “significantly relieved.”

##### DeQi intensity scale

The Chinese Modified Massachusetts General Hospital Acupuncture Sensation Scale(C-MASS) will be employed to evaluate the intensity of diverse needling sensations experienced during acupuncture manipulation. The deqi sensations include: deep aching, sharp pain, pressure, heaviness, distension, tingling, numbness, dull pain, warmth, coldness, and pulsation. The intensity of the deqi sensation is rated on a scale of 0 to 10, 0 indicates no sensation, 1 to 3 is mild, 4 to 6 is moderate, 7 to 9 is severe, and 10 is intolerable intensity. The assessment will be conducted subsequently, immediately upon completion of the acupuncture treatment session.

### fNIRS data acquisition

**Figure 3:**
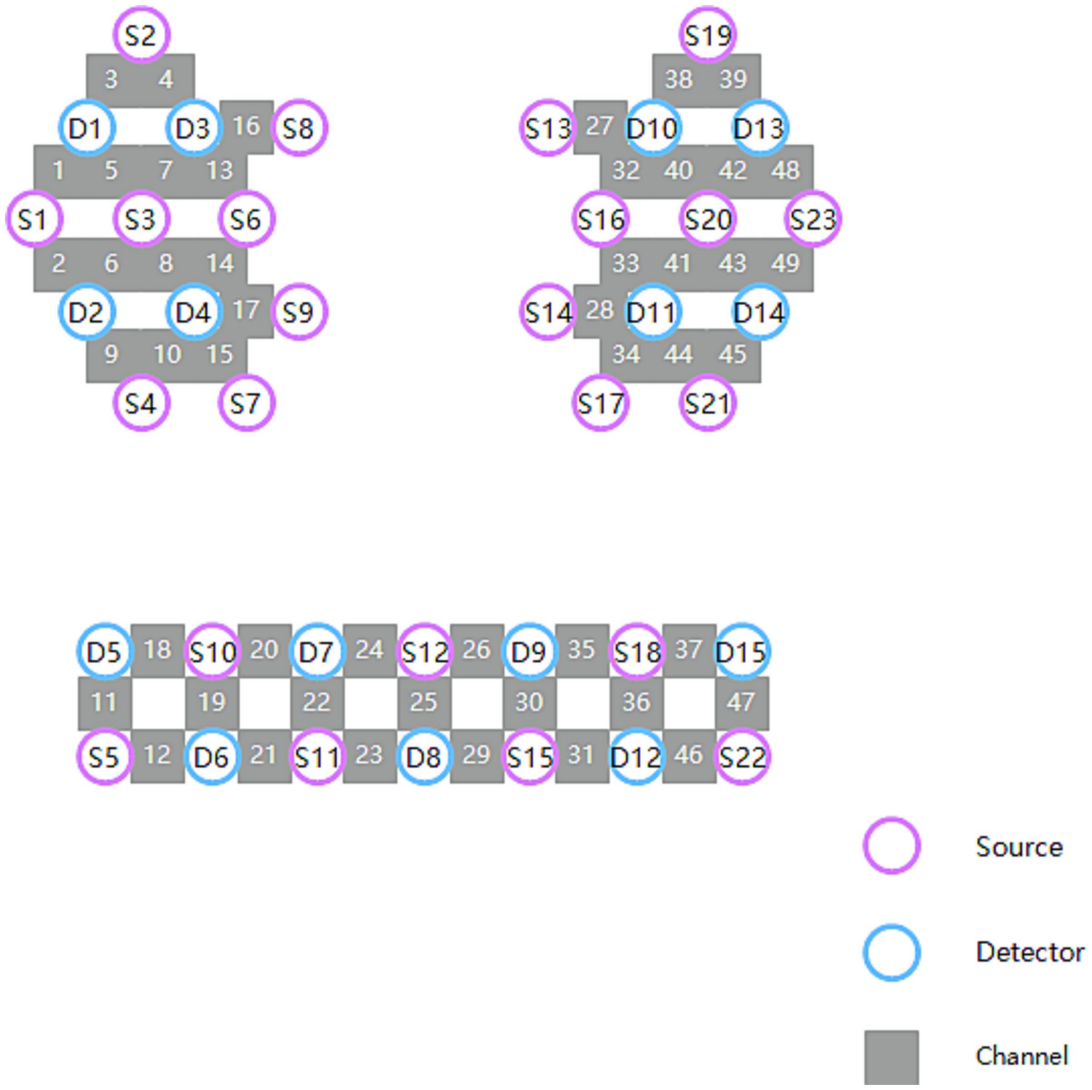
The fNIRS system distribution map.

In this study, a 49-channel fNIRS system (NirSmartII-3000A, NirScan, Danyang Huichuang Medical Equipment Co., Ltd., China) will be used to monitor hemodynamic changes in the prefrontal cortex and motor areas. The sampling rate is 11 Hz, and the appropriate wavelengths for the NIR laser are 730 nm and 850 nm, respectively. The fNIRS detection cap consists of 23 light sources and 15 detectors, with a spacing of 3 cm between the light sources. The channels in the right hemisphere are CH1-CH25; the channels in the left hemisphere are CH26-CH49. The positioning information of the channel, light source and detector is shown in Figure 3.

#### fNIRS task

**Figure 4:**
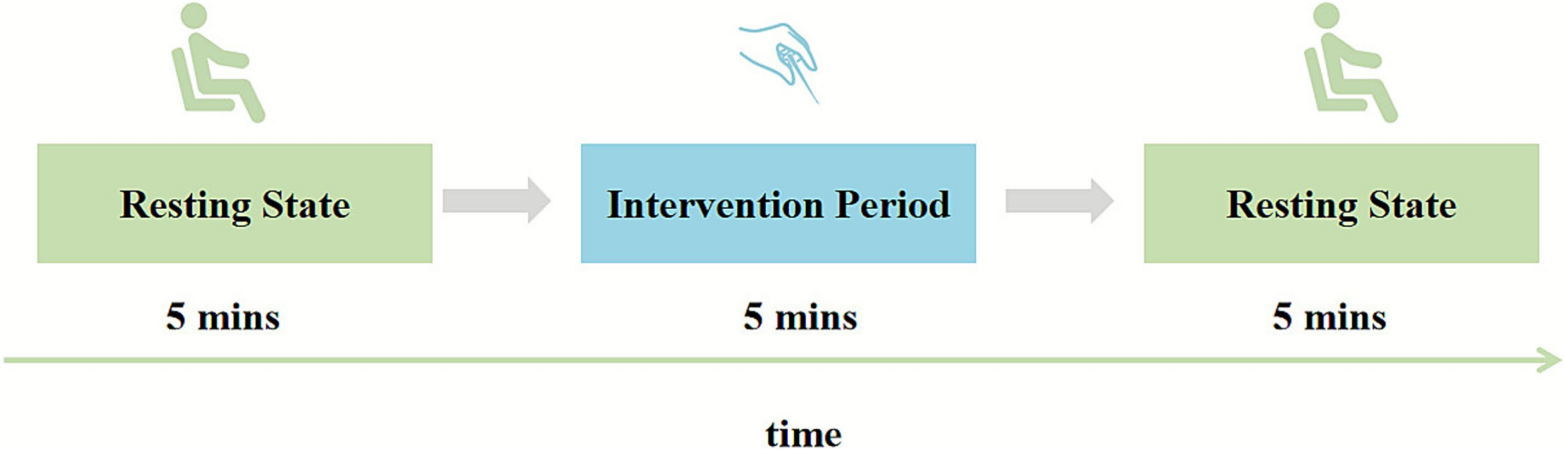
Diagrams of experimental timing.

The fNIRS data will be continuously acquired from participants in a seated position across three consecutive 5-min epochs: (1) pre-acupuncture resting state (5 min before needle insertion), (2) intervention period (during 5-min needle retention), and (3) post-acupuncture resting state (5 min immediately after needle withdrawal). In this study, the term “fNIRS task” refers specifically to the intervention period. Throughout all three phases, participants must remain still and rest with their eyes closed, refraining from speech and minimizing head movement (Figure 4).

#### Data preprocessing of fNIRS

Preprocessing will be performed using the NirSpark system (Danyang Huichuang Medical Equipment Co., Ltd., China). First, channel quality check will be conducted. Second, raw light intensity signals will be converted to optical density (OD) units. Third, motion artifacts will then be corrected using spline interpolation. Fourth, the processed OD data will then be filtered to attenuate physiological noise. Finally, HbO concentration changes will be derived from the filtered data using the modified Beer–Lambert law.

#### Activation analysis of fNIRS

The preprocessed hemodynamic data will be subjected to a standard activation analysis to identify brain regions exhibiting significant task-related responses. The final model specifications and statistical thresholds will be determined based on the data quality and standard practices in the field.

#### Functional connectivity of fNIRS

Functional connectivity (FC) is evaluated by calculating the temporal sequence correlation between different brain regions. The specific metric for quantifying FC (e.g., Pearson’s correlation, phase-based connectivity) and the approach for statistical validation (e.g., Fisher’s z-transformation, permutation testing) will be selected based on the properties of the final processed data.

### Expectation and credibility

Before the first treatment session, the expectation questionnaire will be used to assess the patients’ expectations. Expectation will be measured using the NRS scale, where 0 indicates complete lack of expectation and 10 signifies the maximum expectation. After the final treatment session, the credibility questionnaire will be used for assessment. The credibility scale assesses acupuncture-induced confidence change in itch relief using a 5-point scale ranging from −2 to 2, where −2 indicates “marked decrease,” −1 indicates “mild decrease,” 0 indicates “no change,” 1 indicates “mild increase,” and 2 indicates “marked increase.”

### Adverse events

In case of any adverse event, researchers should immediately stop intervention, take appropriate emergency measures, and meticulously record the time, location, symptoms, severity, handling measures, and outcome of the event. The researchers will examine the causes and evaluate the causal relationship with the intervention.

### Statistical analysis

All statistical analyses will be performed using the IBM SPSS Statistical software package version 26 (IBM, Chicago, IL, USA). All the numerical data will be presented in the form of average values and standard deviations, and will be analyzed using the independent t-test or Mann–Whitney U test. The categorical data will be expressed as number (n) and percentage (%), and the differences between groups will be compared using the Chi-square. The preprocessing and analysis of fNIRS data will be carried out using NirSpark (Danyang Huichuang Medical Equipment Co. Ltd., China).

A two-sample *t*-test will be conducted to compare the brain activation and functional connectivity between the verum acupuncture and the sham acupuncture groups. All statistical tests will use a significance level of 0.05, with corrections for multiple comparisons.

### Data management and quality control

Standardized training shall be implemented for all researchers to ensure comprehensive understanding of the study objectives and protocol requirements. All researchers are required to achieve comprehensive proficiency in the study protocols, including the application of diagnostic criteria, implementation of treatment protocols, execution of randomization procedures, administration of standardized acupuncture techniques, and proper utilization of assessment tools. All the acupuncturists involved in this project have obtained the physician qualification certificates and have received standardized training. Uniformly printed Case Report Forms (CRFs) shall be used to standardize all research documentation. Protocol adherence will be rigorously maintained to guarantee the timely, objective, and accurate completion of all CRFs. Upon review and approval by the quality inspector, the document shall be transferred to the designated data entry clerk for input and storage.

## Discussion

Chronic itch is a common clinical symptom in skin diseases (36, 37). Emerging evidence suggests that chronic itch leads to neuroplastic changes in the brain through sustained abnormal neural activity (21). These alterations are primarily observed as variations in gray matter volume or density within key brain regions (25, 38).

Acupuncture, considered a complementary and alternative therapeutic modality, has been widely employed to alleviate chronic itch. Quchi (LI11) has the effects of eliminating heat, detoxifying, dispelling wind, and relieving itching, as well as harmonizing qi and blood. Electroencephalographic analysis revealed that acupuncture at the LI11 acupoint induced a decrease in alpha frequency in the central region, left parietal lobe, left temporal lobe, and left frontal lobe of healthy subjects (39). The study by Park et al. revealed that acupuncture administered at the LI11 acupoint produced both preventive and therapeutic effects, significantly ameliorating MC903-induced atopic dermatitis-like skin lesions and concomitant chronic itch in mice (40). Napadow et al. used fMRI to demonstrate that acupuncture modulates neural activity by reducing itch-induced activation within key brain regions, including the insula, putamen, premotor cortex, and prefrontal cortex (41). Among these regions, the prefrontal cortex is central to the affective and cognitive dimensions of itch, the pruritic signal is transmitted horizontally across levels in the central nervous system through the “parabrachial nucleus - central medial thalamic nucleus – mPFC” pathway (42). The premotor and motor cortices, as the executive center for planning and executing scratching movements, not only drive the motor response but also encode reward-related signals from movement outcomes, supporting the adaptive regulation of scratching behavior (43).

Currently, neuroimaging research on acupuncture’s effect remains limited, particularly regarding the neural mechanisms underlying acupuncture treatment for chronic itch, which have not been systematically investigated. The exploration of immediate anti-pruritic effects of acupuncture may help reduce potential confounding factors from cumulative treatment effects and provide insights into acupuncture’s acute neuromodulatory responses. Given this knowledge gap, there is a need for exploratory studies to provide preliminary insights into the immediate neuromodulatory effects of acupuncture on core brain regions implicated in chronic itch.

fNIRS offers complementary advantages for intervention studies, including high temporal resolution, portability, and reduced susceptibility to motion artifacts and metal implants, providing significant advantages in clinical applications (30). Its ability to reliably monitor the hemodynamic activity of the superficial cortex makes it ideally suited to capture the dynamic neural responses within the prefrontal and motor regions during acupuncture treatment for itch.

This pilot study employs fNIRS to simultaneously evaluate acute clinical responses and underlying neural mechanisms, providing initial evidence for acupuncture’s potential regulatory effects on central itch processing pathways. Specifically, the present study focuses on the immediate (short-term) neural effects of a single acupuncture session, as captured in real time by fNIRS during treatment. This design enables us to identify transient cortical hemodynamic changes that accompany rapid symptom relief, thereby offering a unique perspective distinct from studies emphasizing long-term or cumulative effects of repeated interventions. By integrating real-time neuroimaging with concurrent clinical assessment, we will achieve preliminary quantification of immediate improvements in patients’ itch symptoms alongside corresponding neural mechanism analysis. Understanding these immediate central mechanisms is crucial for elucidating how acupuncture exerts its antipruritic effect at the onset of treatment, before chronic adaptations occur.

Several limitations should be acknowledged. First, the tactile nature of needling precluded blinding of acupuncture practitioners, potentially introducing performance bias despite rigorous assessor blinding. Second, as a pilot investigation focused on immediate effects, this study will not assess long-term therapeutic outcomes or provide comprehensive exploration of underlying neural mechanisms. Third, due to the limited coverage of the neuroimaging equipment, the acquired data will be restricted primarily to the prefrontal cortex and motor-related areas. Fourth, although fNIRS has lower spatial and temporal resolution compared to fMRI and Electroencephalogram/Magnetoencephalography, its practical advantages—portability, motion tolerance, and compatibility with acupuncture procedures—make it the optimal choice for investigating immediate cortical responses in naturalistic settings.

## Conclusion

As this paper presents a study protocol rather than completed results, our work aims to explore the immediate neural mechanism of acupuncture in treating itch based on fNIRS, providing a scientific basis for the precise diagnosis and treatment of chronic itch.

## Data Availability

The original contributions presented in the study are included in the article/supplementary material, further inquiries can be directed to the corresponding authors.
